# Unexpected presentation and surgical salvage of transplant renal artery dissection caused by vascular clamping: a case report

**DOI:** 10.1186/s12882-020-1699-x

**Published:** 2020-01-29

**Authors:** Shunta Hori, Tatsuo Yoneda, Mitsuru Tomizawa, Kazuki Ichikawa, Yosuke Morizawa, Yasushi Nakai, Makito Miyake, Kiyohide Fujimoto

**Affiliations:** Departments of Urology, Nara Medical University, 840 Shijo-cho, Kashihara, Nara 634-8522 Japan

**Keywords:** Doppler ultrasonography, Kidney transplantation, Transplant renal artery stenosis, Transplant renal artery dissection

## Abstract

**Background:**

Transplant renal artery dissection is a rare and serious event that can cause allograft dysfunction and activation of the renin–mediated renovascular hypertension. Most cases are induced by percutaneous transluminal angioplasty, arteriosclerotic disease, or fibromuscular dysplasia. We observed a case of transplant renal artery dissection induced by unusual causes during kidney transplantation.

**Case presentation:**

A 35-year-old woman, whose mother donated a kidney to her, underwent ABO-incompatible living kidney transplantation. The allograft had one renal artery and vein that were anastomosed to the internal iliac artery and external iliac vein, respectively. Although careful handling was performed in all procedures including vascular clamping, Doppler ultrasonography (US) immediately after reperfusion showed an increase in the systolic blood velocity and urine output was not observed. Arterial anastomotic stenosis was suspected, but upon exploration, a renal artery dissection was detected in the middle portion of the donor artery. The part of the transplant renal artery was resected, and cold reflux was started again. At the resected part of transplant renal artery, dissection was identified. After re-anastomosis, Doppler US revealed that the blood flow of the renal artery was adequate without an increase in the systolic blood velocity, and sufficient blood flow was observed throughout the allograft. Urine output was also observed as soon as blood flow returned, and serum creatinine level decreased to 0.95 mg/dL after surgery. The cause of injury might have been vascular clamping in order to drain the air and check bleeding at the anastomosis.

**Conclusions:**

Our case reaffirmed that careful handling is needed in all procedures, including donor nephrectomy, cannulation for transplant perfusion, vascular clamping, and anastomosis, even without any evidence of arteriosclerosis. Kidney transplant recipients commonly have atherosclerosis and hypertension, which are risk factors for arterial dissection. Early diagnosis and intervention can lead to the prevention of allograft dysfunction. Therefore, close monitoring of allograft blood flow by Doppler US during surgery should be considered.

## Background

Kidney transplantation can be radical treatment for patients with end-stage renal disease (ESRD) and can improve quality of life and survival rates. Despite advanced management strategies such as immunosuppressant therapy, treatment regimen, and surgical techniques, perioperative complications are sometimes experienced. The occurrence rate of vascular complications is around 2–3%, and vascular complications can be a devastating, resulting in allograft loss and allograft nephrectomy [1, 2]. Transplant renal artery dissection (TRAD) is a rare and serious event that can cause allograft dysfunction and activation of the renin–mediated renovascular hypertension [3, 4]. In Japan, kidney transplantation recipients often have a long history of dialysis and systemic arteriosclerosis including the iliac artery. Surgeons are warned not to induce iliac artery dissection during vascular clamping for anastomosis. In the present case, although vascular clamping was performed carefully such that the arteries were not injured, TRAD occurred unexpectedly in a transplanted renal artery. Furthermore, the importance of close examination by Doppler ultrasonography (US) during surgery was reaffirmed to diagnose and perform appropriate interventions as soon as possible for salvage of allograft function.

## Case presentation

**Fig. 1:**
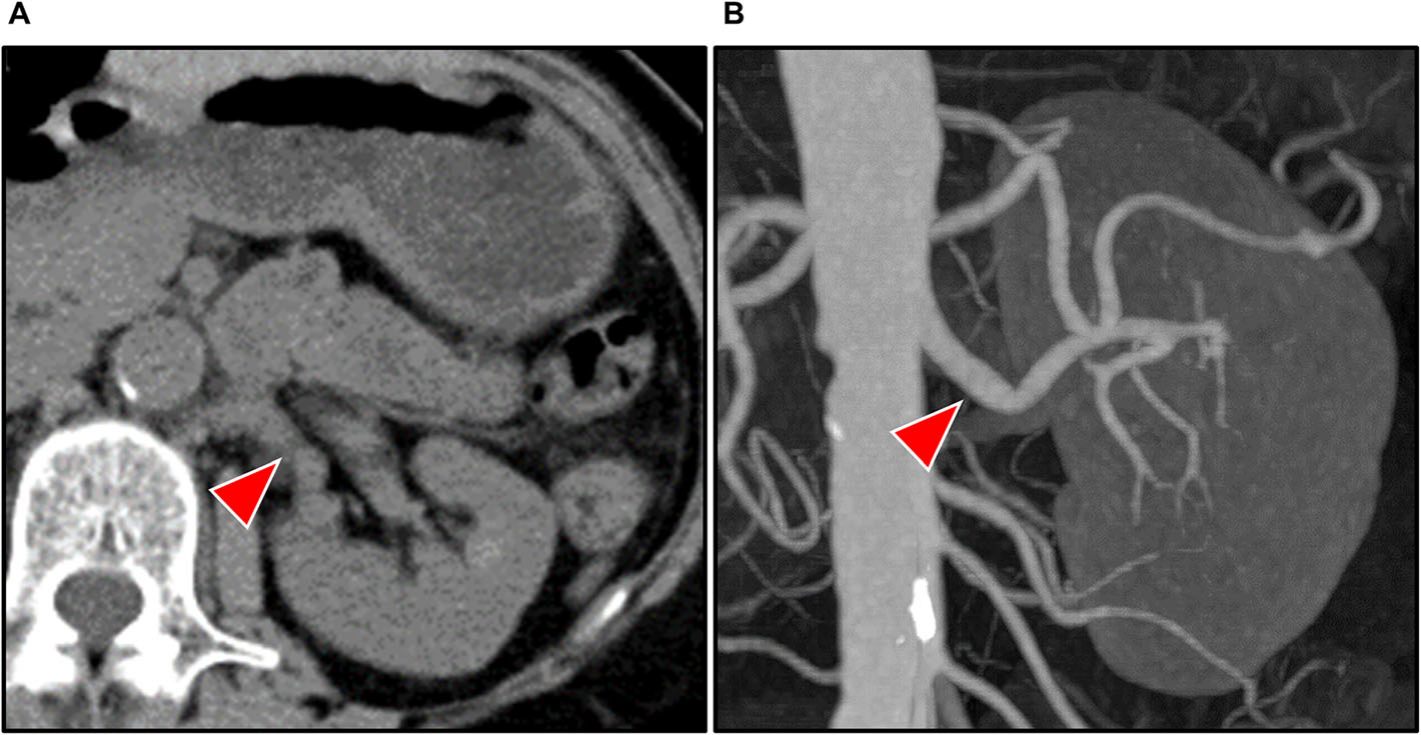
Representative images of the allograft obtained by presurgical unenhanced computed tomography. The donor was the recipient’s mother who was 62 years old, who was good in shape and had no complication. Her left renal artery shows no evidence of arteriosclerosis (red arrow: **a** axial image; **b** three-dimensional image)

**Fig. 2:**
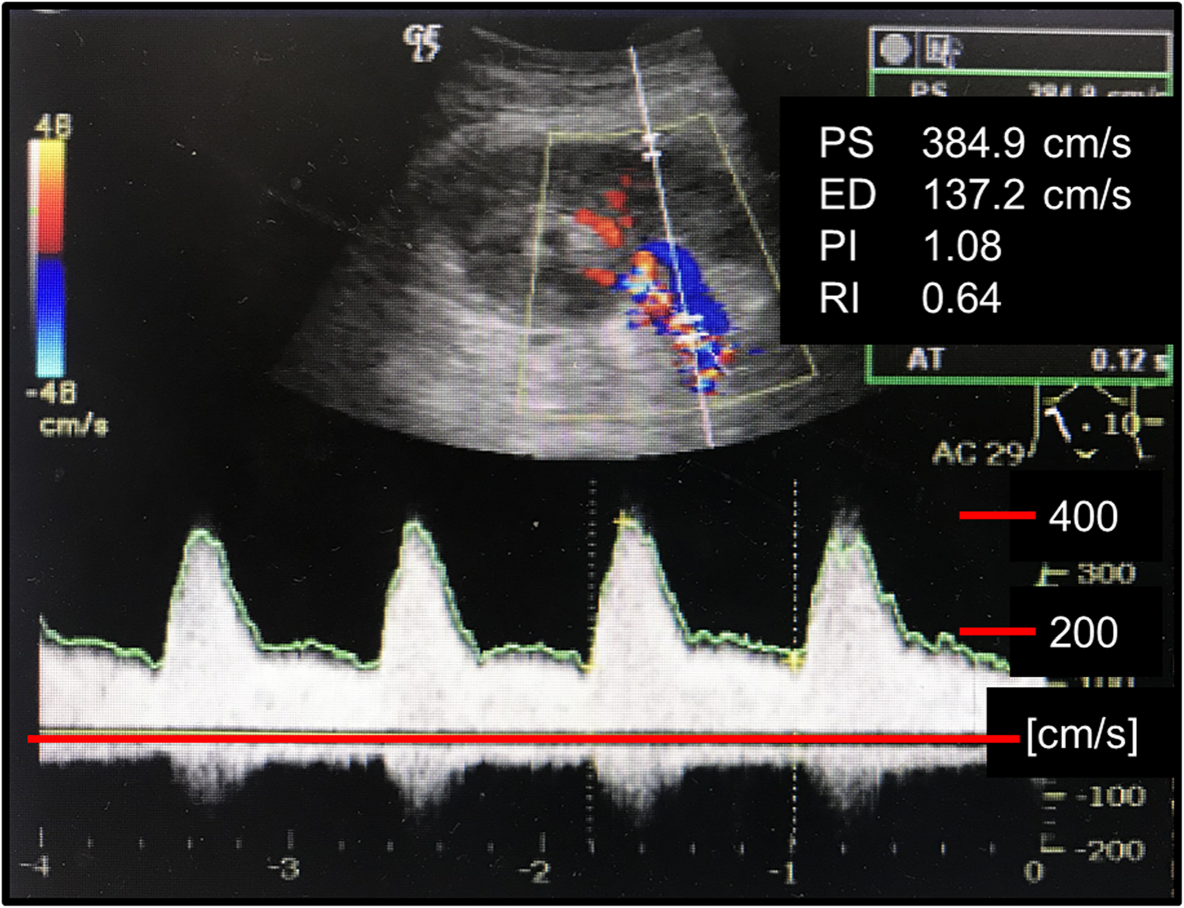
Representative image of the arterial anastomotic stenosis obtained by Doppler ultrasonography. This is a representative image for illustrative purposes. Arterial anastomotic stenosis shows an increased systolic in the systolic blood velocity

**Fig. 3:**
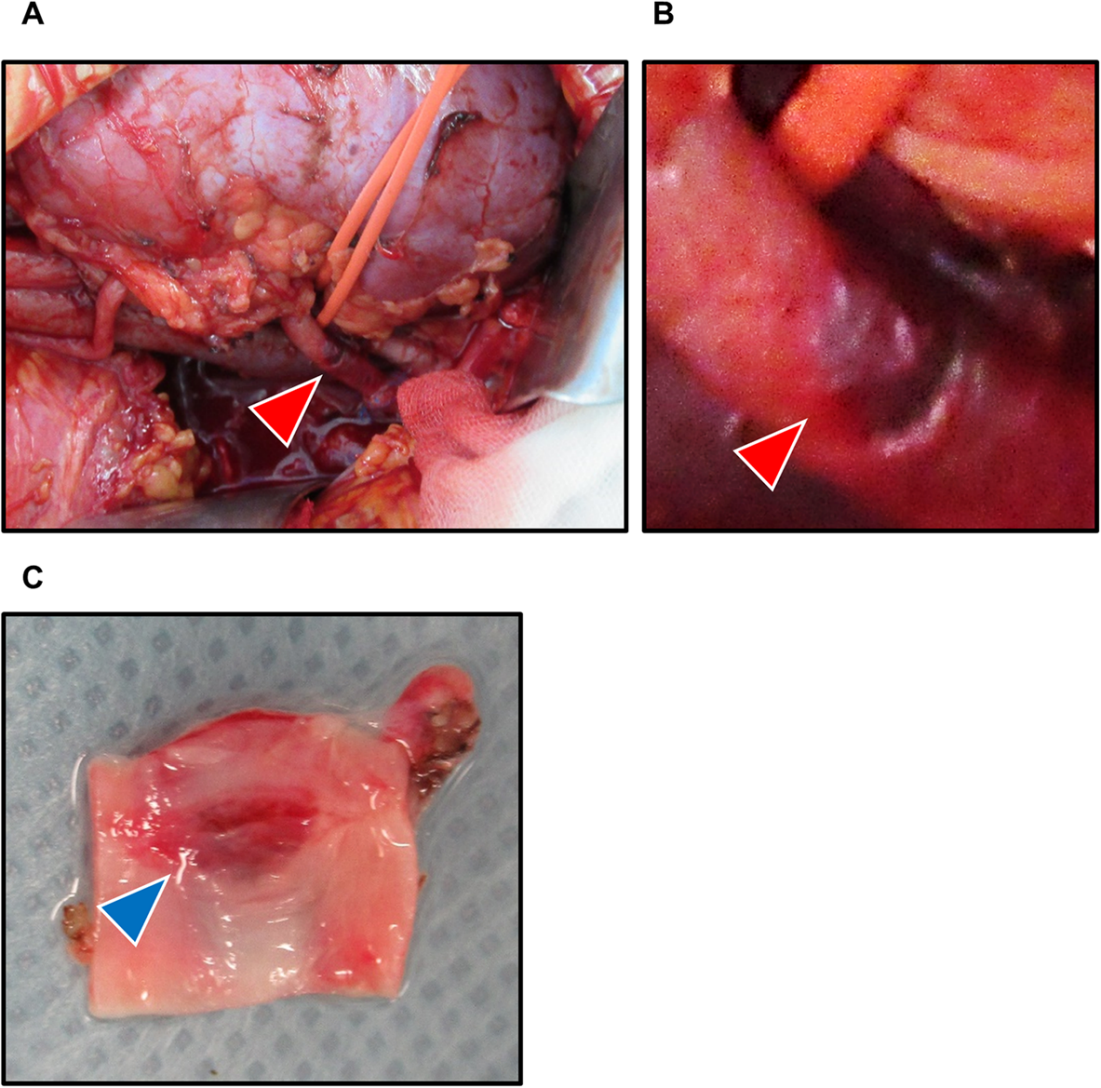
Representative macroscopic images of the allograft and injured transplant kidney artery. Immediately after vascular anastomosis (internal iliac artery-renal artery, external iliac vein-renal vein), Doppler ultrasonography shows an increase in the systolic blood velocity; thus, arterial anastomotic stenosis was suspected and explored. As a result, a color change is observed at the transplanted renal artery (red arrow). The cause of transplant renal artery stenosis was artery dissection (**a** whole image; **b** magnified image). Resected transplant renal artery was cut vertically, and artery dissection was observed macroscopically (blue arrow: **c**)

**Fig. 4:**
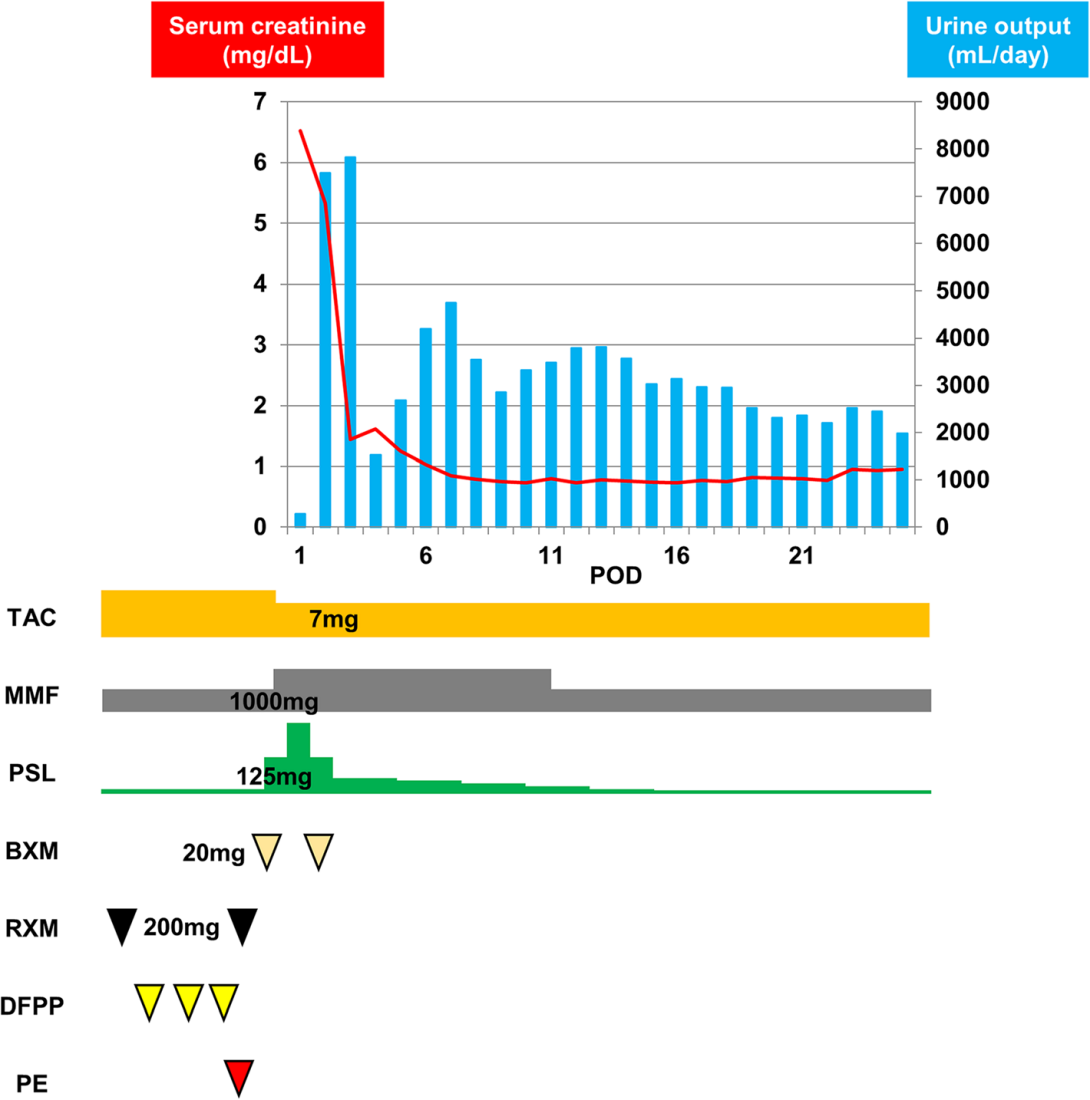
Clinical course of serum creatinine level and urine output and the immunosuppression regimen. After surgery, the serum creatinine level decreased to 0.95 mg/dL, and urine output increased. The immunosuppression regimen was as follows: rituximab, tacrolimus, mycophenolate mofetil, prednisone, and basiliximab. To decrease existing antibody double filtration plasmapheresis and plasma exchange were performed in a presurgical state. At discharge, doses of each immunosuppressive agent were as follows: 7 mg/day tacrolimus, 1000 mg/day mycophenolate mofetil, and 5 mg/day prednisone. On the day of transplantation and postoperative day 4, 20 mg/day basiliximab was administered. POD = postoperative day; TAC = tacrolimus; MMF = mycophenolate mofetil; PSL = prednisone; BXM = basiliximab; RXM = rituximab; DFPP = double filtration plasmapheresis; PE = plasma exchange

A 35-year-old woman who underwent peritoneal dialysis for 11 months because of ESRD secondary to chronic glomerulonephritis was hospitalized for living kidney transplantation. Proteinuria and renal dysfunction were observed during her pregnancy, and her serum creatinine level was 1.4 mg/mL at that time; thereafter, she was followed up by a nephrologist at our institution. Although renal biopsy was considered, the atrophic change of her kidneys was too severe for a renal biopsy for pathological diagnosis. She underwent ABO-incompatible living kidney transplantation donated from her 62-year-old mother. Her left kidney was procured, and the allograft had a single artery that showed no evidence of arteriosclerosis or stenosis (Fig. 1). The transplanted artery was anastomosed to the internal iliac artery, and the transplanted vein was anastomosed to the external iliac vein. After the completion of anastomosis, Doppler US revealed an increased peak systolic flow velocity at around 250 cm/sec with > 200 cm/sec peak velocity at anastomosis correlating with significant stenosis (Fig. 2). Arterial anastomotic stenosis was suspected; however, there was no evidence for it. At the same time, a change in hue was detected in a part of the transplant renal artery; that part of the artery turned dark brown, and hematoma was strongly suspected (Fig. 3). Furthermore, that part was exactly where vascular clamping was performed temporarily in order to drain the air and check bleeding at the anastomosis. Therefore, transplant renal artery stenosis (TRAS) might have resulted from TRAD. The part of the transplanted renal artery was resected, and cold reflux was started again. Injury of the transplant artery was detected macroscopically, and the rest of the transplanted renal artery was anastomosed to the external iliac artery. After re-anastomosis, Doppler US revealed that the blood flow of the renal artery was adequate without an increase in the systolic blood velocity, resulting in sufficient blood flow throughout the allograft. Urine output was also observed as soon as the blood flow returned. An hour after the allograft blood flow returned, the allograft biopsy was performed at the lower pole of the allograft, and no bleeding from the operative field, including the biopsy site, was observed. An immunosuppression regimen including tacrolimus, mycophenolate mofetil, prednisone, and basiliximab was prescribed. After the kidney transplantation, her serum creatinine level decreased to 0.95 mg/dL (Fig. 4). An allograft biopsy showed no evidence of rejection or acute tubular necrosis. Furthermore, pathological diagnosis of the resected artery was tunica media dissection.

Written informed consent was obtained from the patient and her mother for participation in this report.

## Discussion and conclusions

Although rare, TRAD can be a devastating complication, leading to allograft dysfunction and hypertension. TRAS is induced by various factors including surgical technique [5], type of allograft, cold ischemic time [6], incidence of rejection, and immunosuppressive regimen [7]. There have been few reports on TRAS caused by TRAD. TRAD is an uncommon condition usually associated with percutaneous transluminal angioplasty, arteriosclerotic disease, and fibromuscular dysplasia [8–10]. Allograft function after TRAD can possibly be saved with appropriate treatment, including surgical revascularization or stent implantation; therefore, early recognition and diagnosis are really important. Although angiography and computed tomography angiogram are available for early and appropriate diagnoses, angiographic agents are harmful to allografts and are not usually prescribed to kidney transplant recipients. Doppler US has less risks of injury to patient and can be used to easily detect perfusion condition throughout the allograft as well as blood velocity levels. A decrease in allograft perfusion and increase in systolic blood velocity are often observed in cases of TRAS. In our institution, around 40 cm/sec of systolic blood velocity measured at interlobar arteries of transplant kidney is considered to be normal range and 200 cm/sec or more of peak systolic blood velocity at the place of anastomosis is strongly suspected the presence of anastomotic stenosis. In this case, although allograft perfusion was observed throughout the allograft, systolic blood velocity was slightly increased (around 250 cm/sec). Arterial anastomotic stenosis or kinking of the artery were suspected but without any evidence. At the same time, we detected slight swelling and a color change of the proximal transplant renal artery. TRAD was strongly suspected, and we decided to cut part of the transplant renal artery and anastomose it to the external iliac artery. The resected artery was injured, and pathological findings showed intimal dissection. Excessive traction of the transplanted renal artery during donor nephrectomy, cannulation for transplant perfusion, or vascular clamping may cause endothelial or intimal injury, resulting in TRAD. In this case, TRAD might have been caused by the vascular clamping of the transplant renal artery, which showed no signs of arteriosclerosis at all. Arterial dissection after vascular surgery may be triggered by damage from vascular clamping. Atherosclerosis and hypertension are risk factors for arterial dissection, and kidney transplant recipients commonly have systemic arteriosclerosis and hypertension [11]. Iliac arteries, especially internal iliac artery, are often arteriosclerosis in kidney transplant recipients and peripheral vascular disease is also often developed in recipients. Most of arterial dissection have been reported in iliac arteries with severe arteriosclerosis [2]. Careful considerations are needed to determine which iliac artery should be used for arterial anastomosis in kidney transplantation. Although in recipients who has long-term dialysis history we commonly use common or external iliac artery for arterial anastomosis, internal iliac artery is used conventionally if there was less evidence of arteriosclerosis. Therefore, we always carefully treat iliac artery such that it is not damaged by surgical procedures. In this case, we reaffirmed that careful treatment was important for not only the iliac artery but also the transplanted renal artery. The procedure of vascular clamping should be performed using a minimum-weight vascular clip in a place where there is minimal arteriosclerosis, and the direction of vascular clamping should also be taken into consideration. These points should be well discussed preoperatively, and a flexible response during surgery is required. Furthermore, Doppler US provided meaningful live information of the allograft in a non-invasive manner, and even minor changes during Doppler US should be made aware during surgery.

Allograft dysfunction caused by TRAD could be improved by appropriate treatment before irreversible damage occurs. In this case, allograft function was identified as soon as the arterial anastomosis was re-tried, and serum creatinine level decreased to around 0.8 mg/dL 1 week after surgery. Doppler US and a confirmation of chronological change of allograft blood supply during surgery can lead to early diagnosis and intervention, resulting in salvage of allograft function. Although the transplanted renal artery was relatively long enough to resect and re-anastomose in this case, TRAD of the transplant renal artery can be an irreparable complication if it occurs into the distal renal artery as it divides in the hilum. Therefore, careful handling is needed in all procedures, including donor nephrectomy, cannulation for transplant perfusion, surgical clamp, and anastomosis. The donor nephrectomy record was reviewed, but there was no evidence of vascular injury. Thus, there may have been a lack of consideration for the surgical clamp even if there was no arteriosclerosis in the donor renal artery. We also should consider the way not to use vascular clamping of renal artery to prevent TRAD when draining the air and checking bleeding.

In our experience, recognition of clamping injury can prevent unnecessary complications, and close monitoring of allograft blood flow using Doppler US during surgery can lead to early diagnosis and interventions.

## Data Availability

Records and data pertaining to this case are in the patient’s secure medical records in the Nara Medical University.

## Abbreviations

ESRD: End-stage renal disease
TRAD: Transplant renal artery dissection
TRAS: Transplant renal artery stenosis
US: Ultrasonography
